# Inferior acute myocardial infarction with anterior ST-segment elevations

**DOI:** 10.1007/s12471-018-1147-8

**Published:** 2018-08-13

**Authors:** L. J. Bouhuijzen, M. G. Stoel

**Affiliations:** Department of Cardiology, Thoraxcentrum Twente, Medisch Spectrum Twente, Enschede, The Netherlands

A 56-year-old woman presented with chest pain for the past 6 h and a clinical picture consistent with an ST-elevation myocardial infarction. A 12-lead electrocardiogram is shown in Fig. 1. Immediate coronary angiography revealed occlusion of a non-dominant right coronary artery (RCA). Successful intervention with a drug-eluting stent was performed. The left-sided coronary arteries showed no significant stenosis.

**Fig. 1:**
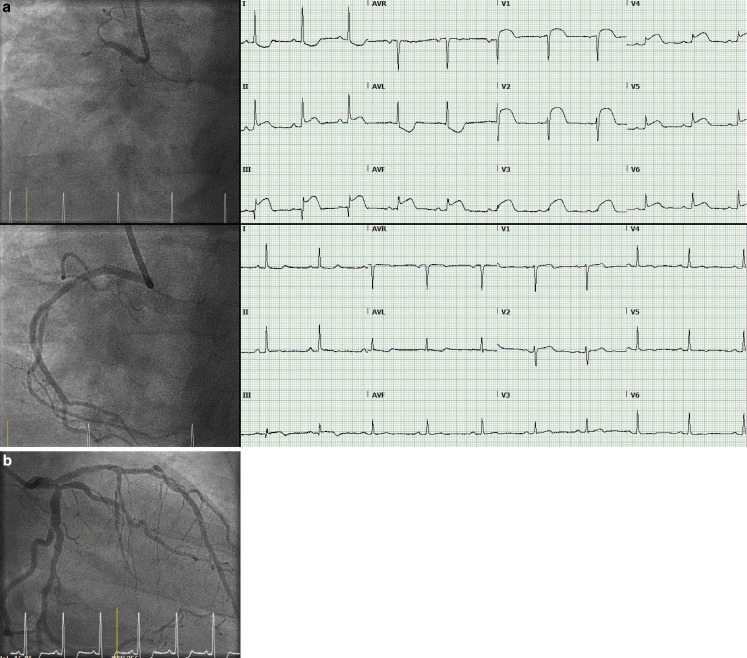
**a** *Above* Coronary angiography of the right coronary artery (RCA) (left anterior oblique 30° view) shows 100% occlusion of the proximal RCA. The corresponding electrocardiogram shows combined anterior and inferior ST-segment elevations. *Below* After percutaneous coronary intervention, electrocardiography showed complete resolution of the ST-segment elevation in all leads. **b** Right anterior oblique view of the left anterior descending and circumflex coronary arteries shows diffuse atherosclerosis without significant stenosis

Acute myocardial infarction with ST-segment elevation in both anterior and inferior leads is usually caused by occlusion of a wrap-around left anterior descending or proximal RCA occlusion [1]. Several mechanisms have been proposed for anterior ST-segment elevation in inferior infarction [2]. In this case, we hypothesised that acute ischaemic right ventricular dilation causes anti-clockwise rotation in the horizontal plane, resulting in ST elevation in all precordial leads. Surprisingly, the presence of concomitant precordial ST elevation is associated with smaller infarct size. This is probably explained by an absence of posterior infarction, which would result in precordial ST-segment depression [3].

## References

[1] Sadanandan S, Hochman JS, Kolodziej A (2003). Clinical and angiographic characteristics of patients with combined anterior and inferior ST-segment elevation on the initial electrocardiogram during acute myocardial infarction. Am Heart J.

[2] Kim SE, Lee J-H, Park D-G (2010). Acute myocardial infarction by right coronary artery occlusion presenting as precordial ST elevation on electrocardiography. Kor Circ J.

[3] Carroll R, Sharma N, Butt A, Hussain KMA (2003). Unusual electrocardiographic presentation of an isolated right ventricular myocardial infarction secondary to thrombotic occlusion of a non-dominant right coronary artery—a case report and brief review of literature. Angiology.

